# New Methodology for Modifying Sodium Montmorillonite Using DMSO and Ethyl Alcohol

**DOI:** 10.3390/ma17123029

**Published:** 2024-06-20

**Authors:** Adriana Stoski, Bruno Rafael Machado, Bruno Henrique Vilsinski, Lee Marx Gomes de Carvalho, Edvani Curti Muniz, Carlos Alberto Policiano Almeida

**Affiliations:** 1 Interfacial Science Laboratory, Department of Chemistry, Midwestern State University, Guarapuava 85040-080, PR, Brazil; adrianastoski@hotmail.com; 2 Department of Chemistry, State University of Maringa, Maringá 87020-900, PR, Brazil; ph54828@uem.br (B.R.M.); ecmuniz@gmail.com (E.C.M.); 3 Group of Biopolymeric Materials and Composites, Department of Chemistry, Federal University of Juiz de Fora, Juiz de Fora 36036-110, MG, Brazil; 4 Department of Chemistry, Federal University of Piauí (UFPI), Teresina 64049-550, PI, Brazil; lee.marx@ufpi.edu.br

**Keywords:** sodium montmorillonite, dimethylsulfoxide, montmorillonite layers, organo-clay, swelling

## Abstract

Modified clays with organic molecules have many applications, such as the adsorption of pollutants, catalysts, and drug delivery systems. Different methodologies for intercalating these structures with organic moieties can be found in the literature with many purposes. In this paper, a new methodology of modifying Sodium Montmorillonite clays (Na-Mt) with a faster drying time was investigated by X-ray diffraction (XRD), Fourier transform infrared spectroscopy (FTIR), BET, and thermogravimetric analysis (TG and DTG). In the modification process, a mixture of ethyl alcohol, DMSO, and Na-Mt were kept under magnetic stirring for one hour. Statistical analysis was applied to evaluate the effects of the amount of DMSO, temperature, and sonication time on the modified clay (DMSO-SMAT) using a 2^3^-factorial design. XRD and FTIR analyses showed the DMSO intercalation into sodium montmorillonite Argel-T (SMAT). An average increase of 0.57 nm for the interplanar distance was found after swelling with DMSO intercalation. BET analysis revealed a decrease in the surface area (from 41.8933 m^2^/g to 2.1572 m^2^/g) of Na-Mt when modified with DMSO. The porosity increased from 1.74 (SMAT) to 1.87 nm (DMSO-SMAT) after the application of the methodology. Thermal analysis showed a thermal stability for the DMSO-SMAT material, and this was used to calculate the DMSO-SMAT formula of Na[Al_5_Mg]Si_12_O_30_(OH)_6_ · 0.54 DMSO. Statistical analysis showed that only the effect of the amount of DMSO was significant for increasing the interlayer space of DMSO-SMAT. In addition, at room temperature, the drying time of the sample using this methodology was 30 min.

## 1. Introduction

Clay materials are abundant, cheap, and considered environmentally green. When intercalated or chemically modified, such materials have attracted significant industry interest due to their interesting properties, such as great specific area, high absorption capacity, catalytic behavior, and small particle size [1]. Thus, many studies targeting intercalation or chemical modification of clay materials have been carried out to use them as nanocomposites [2,3,4], catalysts [5], adsorbents [6,7,8], and drug delivery systems [9]. Montmorillonite (Mt) is a mineral from the 2:1 layered phyllosilicate group [10]. Each Mt layer comprises two tetrahedral sheets containing Si^4+^ and a central octahedral sheet containing Al^3+^. These sheets are held together by oxygen atoms from the crystalline unit cell of the mineral [11].

The interlayer space of these phyllosilicates is approximately 1 nm, and the cations can undergo isomorphous substitutions [12]. Si^4+^ can be exchanged for Al^3+^ in tetrahedral sheets, while in octahedral ones, the Al^3+^ ions can be exchanged for Mg^2+^ or Fe^2+^ [2,11,13]. When these substitutions for less charged cations occur, the structure is no longer neutral and becomes negatively charged [10]. To keep the neutral structure of Mt with such negatively charged layers, there are spaces between the layers where alkaline or alkaline-earth metal cations are present [14]. Mt stands out in composites and nanocomposites due to this configuration. Nonetheless, modifications are necessary in its structure to improve the adhesion of an organic compound on its surface.

Studying this organic–inorganic interface is of great importance in manufacturing new nanostructured materials. Some methods exist to increase the Mt interlayer space to improve dispersion and interaction with organic groups. Some important methods for this purpose are described below.

The silanization technique involves a reaction between the mineral structure’s organosilane and the hydroxyl groups [12,15]. Romanzini et al. [14] studied Mt modification using organosilane in both anhydrous and hydrated alcoholic conditions. They noticed that when using only ethanol as a solvent, the interlayer space increased from 2.4 Å to 3.0 Å; while using hydrated alcohol solution, they obtained an interlayer space of 3.5 Å. Organophilization is another method of cation exchange from the clay galleries [2,12,16,17]. Bee et al. [15] used a quaternary ammonium salt and hydrous ethanol as the solvent, achieving 0.51 nm in the interlayer distance. There is also the swelling technique, which uses polar organic solvents. In this method, the mechanism to modify the interlayer space can occur in two ways: (i) by ion–dipole forces between the cation and the used solvent and (ii) by the coordination complexes. The swelling increases when polar solvents of higher dipole moments are used [18,19]. Nonetheless, the adsorption of amines is more effective than that of alcohols, although the alcohols have a higher dipole moment [19].

Dios-Cancela et al. [19] studied the adsorption of polar organic solvents such as acetone, acetonitrile, trimethyl phosphate, and dimethyl sulfoxide into Mt, which contains different cation types in the interlayer space. The results obtained by Dios-Cancela et al. [19] showed that only the sodium Mt had the d_001_-value altered when the type of the cation was changed. Furthermore, it was observed that when dimethyl sulfoxide (DMSO) was used as a solvent, the interlayer space of the sodium Mt increased by 0.4 nm from the initial spacing. However, the most significant increase of 0.58 nm was found using trimethyl phosphate instead of DMSO. The theoretical results using DMSO as a solvent showed that it can penetrate the clay mineral in different positions, causing an increase (from 0.46 to 0.54 nm) in the interlayer space, forming only one layer of this solvent in the Mt gallery.

Intercalation of Mt with small organic molecules, such as DMSO, changes its structure from hydrophilic to hydrophobic and reduces the surface tension between the lamellae, facilitating its interaction with organic compounds, thus providing various applications for this new material [20,21].

Water is another substance that can be used to swell clay lamellae [22]. For example, Amorim et al. [23] studied the swelling process of three types of clay through the intercalation of water molecules in a saline medium. Furthermore, water, coordinated with exchangeable cations, plays an important role in the interaction of Mt with small uncharged organic compounds [24,25]. The polar groups of these compounds compete for the same binding sites around the cation as water, which assists in the process of attaching uncharged multifunctional organic polymers to the clay surface. Constanza-Robinson et al. [26] used water to cause swelling of the lamellae and then added a macromolecule to increase the interlamellar space. However, in these studies [23,26] it took 24 h for the process to be completed. 

Additionally, the intercalated compounds of Mt with small molecules are generally prepared in an open reactor at a certain temperature, and this process can take a long time, usually between 12 h and 48 h [13,19,27]. However, there are cases where it took 4 days for the reaction to take place [28].

This current study proposes a new methodology for modifying sodium Mt using a mixture of DMSO and ethyl alcohol (EA). The product has been characterized by X-ray diffraction (XRD), Fourier transform infrared spectroscopy (FTIR), thermogravimetric analyses (TG and DTG), and BET. In addition, statistical analysis has also been applied for evaluating the different treatments. The primary purpose is to reveal better conditions to obtain Mt clay for use in other applications, using a minimum reaction time compared to the above-mentioned methodologies.

## 2. Materials and Methods

### 2.1. Materials

The clay used in this study was sodium montmorillonite (Na-Mt) Argel-T (SMAT) provided by Buntech Tecnologia em Insumos LTDA—São Paulo/SP, Brazil, with the following chemical composition: SiO_2_ 63.0%, Al_2_O_3_ 18.0%, Fe_2_O_3_ 4.0%, MgO 3.0%, CaO 2.0%, Na_2_O 2.0%, and TiO_2_ < 1.0% [29]. The methylene blue titration method determined the cation exchange capacity of 118.74 meq/100 g. SMAT has been standardized with particle sizes smaller than 100 mesh (<0.149 mm).

The organic solvents used were DMSO (99.9%), supplied by Dinâmica^®^ (Indaiatuba-Brazil), ethyl alcohol (EA 99.5%), provided by Neon^®^ (Suzano-Brazil), and ethyl ether (99.9%), supplied by Anidrol^®^ (Diadema-Brazil). All chemicals used in this project were of analytical laboratory grade.

### 2.2. SMAT Modification with Polar Organic Solvents

SMAT was modified at atmospheric pressure in a round-bottom 100 mL glass reactor equipped with a magnetic stirrer, thermometer, and reflux condenser. This system was used in all experiments with the following methodology: SMAT (1 g), DMSO (1, 3, or 5 mL), and EA (20 mL) were added in the glass reactor, and the system was heated to the required temperatures (20, 40, or 60 °C). After a reaction time of 1 h, the heat was turned off, and the mixture was filtered through a white band filter paper. The obtained solid phase was sonicated with 10 mL of ethyl ether for 0, 90, or 180 s to evaluate the influence of sonication in the modification. The mixture was filtered through a white band filter paper again, and the solid was dried at room temperature until it reached a constant mass. The modified clay was called DMSO-SMAT.

### 2.3. Factorial Design Studies

The effects of the amount of DMSO, temperature, and sonication time were investigated through a 2^3^-factorial design, considering the basal reflection (d_001_-value) distance on DMSO-SMAT as the analyzed response to understand the best conditions to achieve an increased interlayer space.

The experiments were performed in duplicate and ran randomly for all level–input combinations, as shown in Table 1.

The effect (*E_f_*) of each input factor of the factorial design for the increase in the SMAT interlayer space was calculated using the following equation:

*E_f_ = R_+_ − R_−_*

where *R_+_* and *R_−_* are the averages of the responses obtained at the superior (+) and inferior (−) levels, respectively.

The statistical significance of the effects of the inputs was evaluated by standard error, confidence intervals, and Pareto chart calculations at 95% confidence. All statistical analyses were performed using the software “Minitab for Windows v.16”.

### 2.4. Characterization of the SMAT and DMSO-SMAT Samples

The XRD measurements were collected using a D2 Phaser diffractometer with Cu K_α_ radiation (λ = 1.5418 Å) between 4° and 40° (2θ) with a scanning rate of 2°/min and a step of 0.07°/s. SMAT and DMSO-SMAT d-spacings were computed by applying the Bragg’s equation, as follows:

(1) sin⁡θ=nλ/2d

where *λ* is the wavelength of the incident X-ray, *d* is the distance between the clay layers, n is an integer, and *θ* is the incident angle. 

FTIR spectroscopy was used to identify functional groups present in the samples. FTIR spectra were recorded on a Perkin Elmer spectrometer using the Attenuated Total Reflectance (ATR) method in the wavenumber range of 4000 to 650 cm^−1^ and at a nominal resolution of 4 cm^−1^ obtained after accumulating 6 scans.

Thermogravimetric (TG) and differential thermogravimetry (DTG) analyses were performed using a thermal analyzer (STA 8000 Perkin Elmer TM). For each sample, 12 mg of SMAT or DMSO-SMAT was placed into an Al_2_O_3_ pan and heated from 40 to 900 °C at a 20 °C/min rate under an inert atmosphere (N_2_).

Specific surface area was obtained by BET N_2_ adsorption–desorption measurements (Micromeritics, TriStar II 3020 model). The results were obtained using pure liquid N_2_ adsorption at 77 K with a PP0 between 0.05 and 0.35. Before the adsorption experiments, the samples were outgassed under vacuum conditions for 12 h at 50 °C.

## 3. Results and Discussion

### 3.1. Analyses of the SMAT and DMSO-SMAT Samples

**Figure 1:**
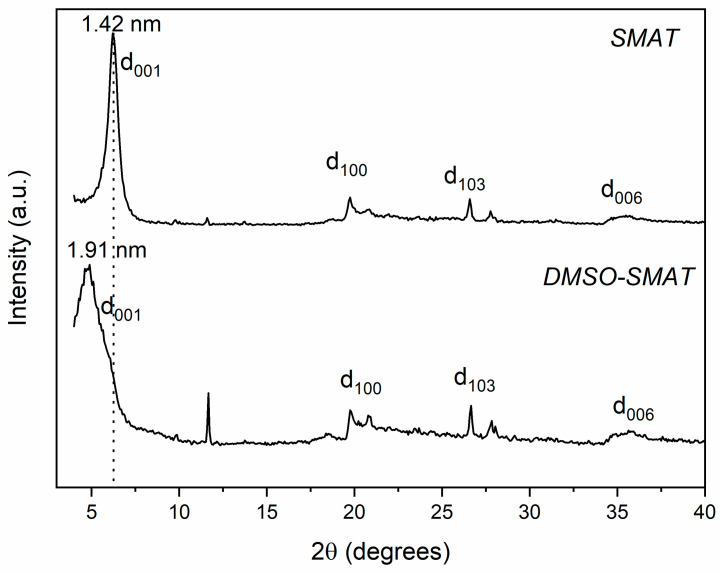
XRD patterns of unmodified Mt (SMAT) and modified Mt (DMSO-SMAT) samples.

X-ray diffraction (XRD) analysis was performed to evaluate the increase in the *d*-value to verify the intercalation of DMSO into the interlayer space of SMAT. Figure 1 shows the XRD patterns of the SMAT and DMSO-SMAT samples. In both cases, the spectrum shows the diffraction peak corresponding to the 001 crystallographic plane, representing the interlayer space. The DMSO-SMAT pattern shows a shift of this diffraction peak toward a lower 2θ value; the peak shifts from 6.21° to 4.62°, proving the intercalation of DMSO in SMAT [29,30] using a reaction time shorter than those reported in the literature [13,19,27], making the use of this methodology feasible.

Through the XRD profiles shown in Figure 1, the interlayer space for the 001 reflection was calculated as 1.42 nm and 1.91 nm for the SMAT and DMSO-SMAT samples, respectively, i.e., there was a lamellar increase of 0.49 nm after applying the methodology with a mixture of DMSO/ethanol solutions. This increase suggests a monolayer intercalation of DMSO in the clay gallery, as verified by Dios-Cancela et al. [19] through a computational study in which the authors found two possible orientations for DMSO when it was used as a modifying agent with an interlayer increase varying from 0.46 to 0.54 nm. The most probable explanation is that the sulphonyl group’s oxygen is oriented toward the center of the cation, reducing the repulsion between this oxygen and the tetrahedral silicate oxygens. Additionally, there is an electrostatic interaction between the negatively charged oxygens of silicate with the positively charged sulfur atom in this group [19].

Castrillo et al. [31] used a mixture of DMSO with distilled water at 40 °C for 71 h under stirring to intercalate DMSO in kaolinite and obtained a lamellar increase of 0.41 nm, thus generating a very similar result to the one obtained in this study, but with a higher reaction time. Castrillo et al. [31] have also shown that there are two possible configurations for DMSO in the interlayer space, promoting lamellar increases of 0.2 nm and 0.4 nm, with the latter being more stable as the S=O groups from DMSO interact with the octahedral layers from the kaolinite through the formation of hydrogen bonds with the hydroxyl groups of the clay surface.

Additionally, Figure 1 shows that the reflection peak for the DMSO-SMAT sample is broader compared to the SMAT one. The literature shows that this behavior may be related to the process of delamination or exfoliation of the clay layers, making the structure more disordered [14,27] and favoring the production of nanocomposites [32]. However, studies about the changes in the interlayer spacing of clays show that an enlarged peak may be related to a process called osmotic swelling, which occurs when water molecules migrate into the clay layer to try to restore the cation balance in the clay lamellae [23,33]. Due to the high interaction between water and DMSO, the organic molecules must migrate together with water molecules and assist in the cation solvation process, as described by Nakato [34]. This assumption is also in agreement with Golova et al. [35], as the water/DMSO system causes the interlayer spacing to be increased due to this migration, evidenced by the shift of the d-001 peak to lower 2θ values. However, in the case of DMSO-SMAT, there is no repulsion similar to that mentioned by Golova et al. [35], which causes the lamellae to shrink, as the enlarged peak shown in Figure 1 has a shoulder at the same 2θ value as SMAT and not in higher values (dotted line) as proposed by the authors.

Beyond the d_001_ diffraction peak, Figure 1 also presents other crystallographic plans at 19.68°, 26.58°, and 35.50°, which indicate the d_100_, d_103_, and d_006_ basal planes, respectively [29,30]. Still, the XRD pattern for modified clay shows a crystalline peak at 11.66°, possibly related to highly uniform intercalated DMSO molecules in the Mt, creating distinct interlayer spaces [26,34].

**Figure 2:**
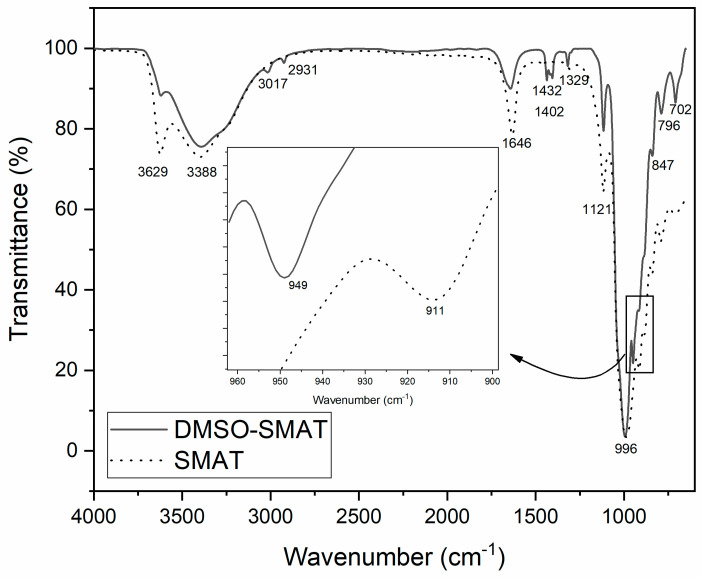
FTIR spectra of modified Mt (DMSO-SMAT) and unmodified Mt (SMAT) samples with emphasis on the region between 960 and 900 cm^−1^.

Fourier transformed infrared (FTIR) analyses of the SMAT and DMSO-SMAT samples showed that DMSO penetrated into the interlayer space, as illustrated in Figure 2. The wavelength bands at 3629 and 1646 cm^−1^ refer to the M-OH and -OH groups of hydration of the clay lamellae [36,37], respectively, showing a decrease in intensity after applying the methodology.

The band at 1121 cm^−1^ is due to the Si-O stretching vibrations. The bands at 996 cm^−1^ and 3388 cm^−1^ are related to the presence of Si-O on the plane and OH stretching hydration, respectively, without changes in the intensity [36,37,38], showing that DMSO intercalation did not interfere with these bonds. The presence of DMSO in DMSO-SMAT is revealed by the 3017 and 2931 cm^−1^ bands due to the asymmetric and symmetric stretching vibration of C-H, respectively [39,40], and by C-S-C asymmetric stretching of DMSO at 706 cm^−1^. The stretching at 1329 cm^−1^, 1402 cm^−1^, and 1432 cm^−1^ of pure liquid DMSO are observed in the DMSO-SMAT sample [38,41]. Bands at 914 and 841 cm^−1^ are also characteristic of SMAT due to the Al-OH-Al and Al-OH-Mg groups, respectively [36,37,38]. The bending mode of the Al-OH is shifted to a higher wavelength due to an interaction with DMSO [41], showing that there is an interaction between the S=O group and the Mt layers.

**Figure 3:**
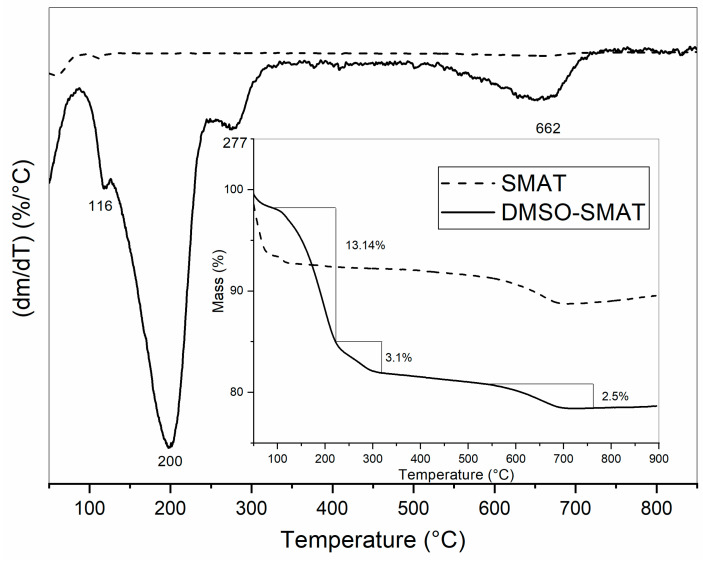
TG and DTG curves for modified Mt (DMSO-SMAT) and unmodified Mt (SMAT) samples.

Figure 3 shows the thermal behavior of the SMAT and DMSO-SMAT samples. Thermal analysis is a valuable technique for obtaining quantitative and qualitative information about the decomposition of the material.

The thermogram of DMSO-SMAT, shown in Figure 3, presents two new stages of mass loss because of the intercalation of DMSO; these are present at 200 °C and 277 °C due to evaporation and decomposition of DMSO [14,42]. An endothermic effect between 80 and 117 °C in both samples can also be observed due to a mass loss of hydration water [19]. For the DMSO-SMAT sample, there were two other endothermic effects, the first one starting at 117 °C and ending at 226 °C with a mass loss of 13.14%, and the other ending at 303 °C with a mass loss of 3.1% due to the DMSO molecules [19,42]. Due to the metal dihydroxylation, there was an endothermic effect between 500 and 717 °C in both samples, with a mass loss of 2.5% [14]. 

From Figure 3, the amount of intercalated DMSO molecules per SMAT unit cell was calculated [43]. As a result, the DMSO-SMAT sample can be represented by the formula Na[Al_5_Mg]Si_12_O_30_(OH)_6_ · 0.54 DMSO. These data demonstrate that DMSO molecules are intercalated into the Mt structure and are responsible for the interlayer increase observed in Figure 1. 

The specific surface areas of the SMAT and DMSO-SMAT were calculated by the Brunauer–Emmett–Teller (BET) method with the relative pressure PPo ranging between 0.05 and 0.35 [44,45]. Significant changes in the BET method were observed after DMSO treatment; the specific surface areas of the SMAT and DMSO-SMAT were found to be 41.8933 m^2^/g and 2.1572 m^2^/g, respectively. This decrease shows that DMSO molecules are in the SMAT interlayer space, reducing the accessibility of nitrogen gas molecules to reach the internal surface of the SMAT [29,44,46]. The results obtained here are in accordance with other research, which has shown a decrease in the surface area of clays after intercalation with organic molecules [29,46,47,48,49]. For example, Leal and coworkers [48] promoted modifying kaolinite with DMSO. BET results revealed a decrease in the surface area from 33.53 to 27.48 m^2^g^−1^ after intercalation. dos Santos and coworkers [29] obtained montmorillonite modified with the cationic surfactant cetyltrimethylammonium bromide (CTAB). The surface area decreased after intercalation, changing from 47.76 to 15.06 m^2^g^−1^. These results revealed that the modified materials presented a greater capacity for the removal of water contaminants, such as p-nitrophenol, etheramine, and heavy metals [29,48,49].

The SMAT average pore size increased from 1.74 nm to 1.87 nm, indicating that DMSO molecules occupied the internal regions of the micropores, reducing their contribution to the total pore volume and leaving only larger diameter pores available [44,45,46]. Leal and coworkers [48] reported that the intercalation of DMSO molecules occurred in the kaolinite interlayers, and as these molecules were bulky enough to increase the interlayer space, this characteristic also explained the pore size increase.

These results reinforce that the broadened peak illustrated in Figure 1 for the DMSO-SMAT sample is related to the swelling process described by Amorim et al. [23] and probably not to the structure delamination, which would increase its surface area.

Finally, the thermal analysis revealed that the SMAT-DMSO material presents thermal stability to be applied to several applications, such as adsorption and drug delivery purposes [45,48].

### 3.2. Factorial Design Study 

In this paper, the methodology used to increase the interlayer space of the Na-Mt was developed and quantitatively evaluated through factorial analysis. The influences of the temperature, amount of DMSO, and sonication time were assessed through a 2^3^-factorial design (Table 2), and the values of the main and interaction effects are shown in Table 3. For a better understanding of the effects, two central point experiments, runs 9 and 10, were used and are demonstrated in Table 2.

The standard error analysis and confidence intervals of 95% showed that only the amount of DMSO used was statistically significant for clay modification (Table 3). The estimated value of the effect for input B (amount of DMSO) was nearly 3.5 times higher than the respective standard error.

Na-Mt modification is independent of the used temperature (input A), and washing the sample with ethyl ether was sufficient to remove the excess DMSO and helps with the drying process because input C was not statistically significant, i.e., there was no influence in the response if using 0 or 180 s for the sonication time, indicating that this factor does not help with the washing process or with the entry of DMSO molecules into the interlayer space.

**Figure 4:**
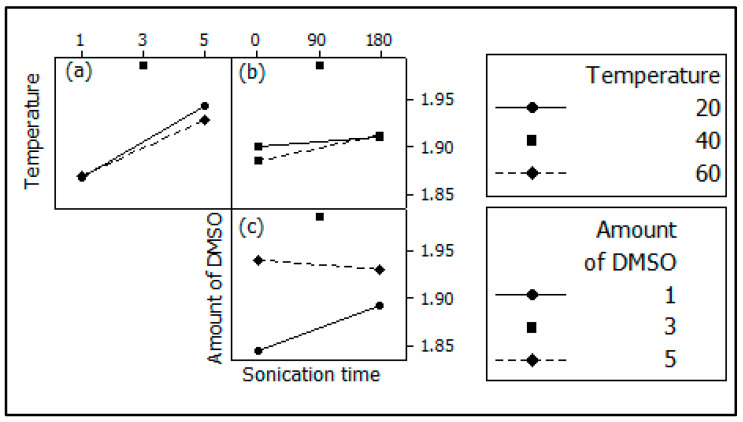
Interaction effects between: (**a**) temperature and amount of the DMSO; (**b**) temperature and sonication time and (**c**) amount of the DMSO and sonication time for the 2^3^-factorial designs for interplanar space increase.

The interaction effects between the studied factors were traced, and the results are shown in Figure 4. Figure 4a shows that the simultaneous change of the temperature and amount of DMSO produced an antagonistic effect because as the values of both inputs increased, the value of interplanar space also decreased. When using 1.0 mL of DMSO, the response was the same for both temperatures. However, when using 5.0 mL of DMSO, there was a slightly higher lamellar space increase at the lowest temperature.

This event occurred because the interaction between DMSO molecules and Mt layers can occur in three distinct spaces: (a) on the outer edges, (b) on the edges, and (c) on the inner surface [50,51]. Thus, as more DMSO molecules are available for interaction and the higher the system temperature, there is less selectivity in the interaction and, consequently, the DMSO molecules interact more at the edges of the Mt layers, reducing the interlayer space. However, by decreasing the system temperature, the interaction becomes more selective, thus there is a higher interaction on the inner surface.

Figure 4b shows a larger interlayer space without sonication at a lower temperature. The responses were equal for both temperatures when sonicating the sample for 180 s. This indicates that when using sonication, DMSO was displaced to the inner surface instead of the edges of the Mt layers.

Sonication time had a higher effect when using 1.0 mL of DMSO instead of 5.0 mL, as indicated in Figure 4c. This may be due to the displacement of DMSO to the inner surface instead of the edges, providing a higher interlayer space (for 1.0 mL). With a higher amount of the DMSO, agitation did not have a significant effect, as there were many molecules reacting on the inner surface, increasing the interlayer space.

It is worth noting that all interactions did not have significant effects at 95% confidence; however, the interaction (B) × (C) had the highest interaction effect, as evidenced by Table 3 and Figure 4c.

In addition, the highest lamellar space increase was in the central point runs, with an average of 1.99 nm, with a real increase of 0.57 nm between the clay lamellae. Thus, 3 mL was the highest amount of DMSO penetrating the interlayer space. Increasing the interlayer space with DMSO addition is essential for several applications, such as environmental and drug delivery systems. For example, Leal and coworkers revealed that DMSO increases the kaolinite interlayer space by 8.01 Å for materials for etheramine removal from mining wastewater [48]. The authors also revealed that DMSO influences the creation of new adsorption sites that were previously inaccessible. The authors discussed that these results promoted an increased selectivity and amount of etheramine removal [48]. Our results revealed that DMSO intercalation changes the surface characteristic of the final material, which can increase its binding with some adsorbates, even with a decrease in the surface area. The modified material can exhibit a superior pollutant removal capacity in future applications [29,45,47,48,49,52].

### 3.3. Drying Method

In addition to the factors already described, the drying time of the sample is worth mentioning. When only DMSO is used as a solvent, drying takes a few days at room temperature or a few hours in a heated oven.

Dios-Cancela et al. [19] used a process under reduced pressure (at 10^−2^ mmHg) to dry the sample of the clay mineral and DMSO until it was at a constant mass. Y. Zhang et al. [53] used centrifugation to separate the suspension from the solvent, and then the sample was washed three times with an organic solvent.

**Figure 5:**
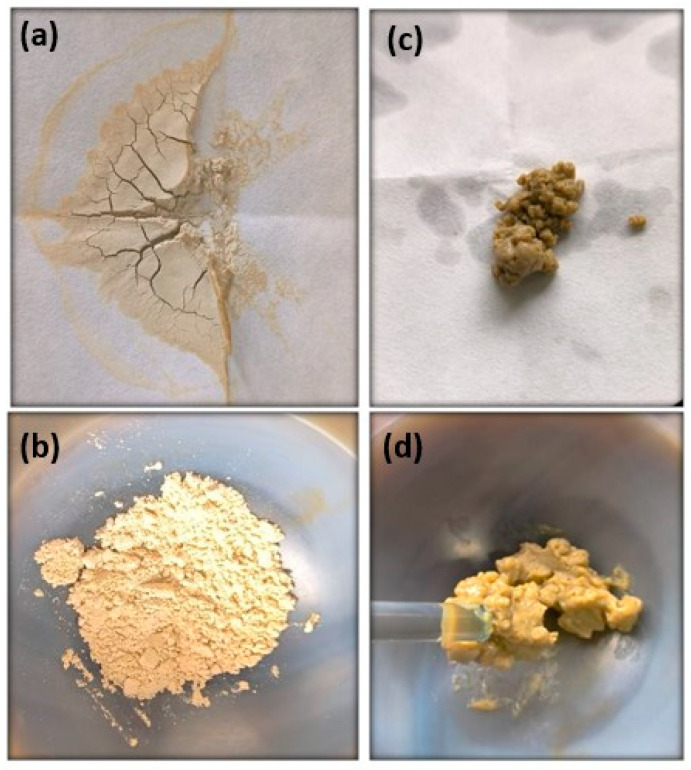
Appearance of the samples using the procedure of Section 2.2: (**a**) 10 min and (**b**) 30 min of drying with ethanol; (**c**) 10 min and (**d**) 30 min without ethanol.

In the process described in Section 2.2, DMSO-SMAT has practically a dry appearance after being washed with ethyl ether and subsequent filtering. However, when removing ethyl alcohol from the procedure, the sample has an entirely different appearance, as shown in Figure 5.

Figure 5 shows that the drying time is short if ethanol and DMSO are added to SMAT. After 30 min at room temperature, the clay mineral was dried and had a constant mass (Figure 5b), whereas in Figure 5d, after the same time (30 min), the sample without ethanol was still very wet.

The sample without ethanol was kept at room temperature for a week and the appearance remained the same as in Figure 5d. In this way, evaporating the excess solvent would only be possible in an oven at a high temperature because DMSO’s boiling point is 189 °C. However, when placing this sample to dry in an oven, some intercalated DMSO molecules would be desorbed, reducing the interlayer space [54]. 

All the information obtained here is important for future researchers to obtain an optimized material for different applications, such as catalysis, drug delivery systems, pollutant removal, etc.

## 4. Conclusions

We evaluated the influences of different factors (DMSO content, sonication time, and temperature) for synthesizing sodium montmorillonite materials intercalated with DMSO in the presence of ethyl alcohol. XRD, FTIR, and BET analyses were successfully used to monitor the intercalation process of DMSO into the interlayer space of SMAT. The DMSO molecules penetrated the interlayer space with the help of the water molecules present in the system, making the DMSO capable of solvating the cations into the interlamellar space, forming hydrogen bonds with the octahedral group of the clay, and, consequently, increasing the interlayer space to 0.57 nm on average. This process further caused the surface area of the clay to decrease significantly, indicating that DMSO molecules have occupied pores from SMAT. However, if certain conditions are used, these averages would decrease because the DMSO molecules may interact with the -OH groups on the side of the SMAT rather than in the interlayer space. 

Furthermore, the use of the proposed methodology drastically reduces the reaction time used for the intercalation of DMSO molecules into SMAT. A reaction time of 1 h is sufficient for intercalation to occur.

The factorial analysis demonstrated that only the amount of DMSO directly impacts the increase in the SMAT interlayer space. Both temperature and sonication time inputs do not present a meaningful interference to the interplanar space of Na-Mt materials.

It was noticeable that adding ethanol to the methodology influenced the drying process of the sample. Our experience revealed that the ethanol sample lasted only 30 min until it dried. Also, it is possible to evaporate the excess solvent using an oven at high temperatures. However, this process leads to the loss of intercalated DMSO molecules, decreasing the interlayer space. It is worth noting that these results are essential to future research in developing similar materials for different applications, such as the development of new adsorbents.

## Figures and Tables

**Table 1:** Factors and levels used in 2^3^-factorial designs as an attempt to increase the values of the interplanar distance (d_001_). Where + and – indicate the superior and inferior levels used in the factorial design, respectively.

Factors	Level (−)	Level (+)
1. Temperature (°C)	20	60
2. Amount of DMSO (mL)	1	5
3. Sonication time (s)	0	180

**Table 2:** The average interplanar space of SMAT used in the 2^3^-factorial design analyses.

Run	Temperature (A) (°C)	Amount of DMSO (B) (mL)	Sonication Time (C) (s)	Interplanar Space (nm)
1	+1 (60)	+1 (5)	+1 (180)	1.93 ± 0.014
2	−1 (20)	+1 (5)	+1 (180)	1.93 ± 0.056
3	+1 (60)	−1 (1)	+1 (180)	1.89 ± 0.021
4	−1 (20)	−1 (1)	+1 (180)	1.89 ± 0.071
5	+1 (60)	+1 (5)	−1 (0)	1.92 ± 0.021
6	−1 (20)	+1 (5)	−1 (0)	1.95 ± 0.021
7	+1 (60)	−1 (1)	−1 (0)	1.84 ± 0.021
8	−1 (20)	−1 (1)	−1 (0)	1.84 ± 0.021
9	0 (40)	0 (3)	0 (90)	1.94 ± 0.064
10	0 (40)	0 (3)	0 (90)	2.03 ± 0.064

**Table 3:** Global mean and input effects and their standard errors for the 2^3^-factorial design to increase the interplanar space.

Parameters	Estimated Value ± Standard Errors
Global mean	1.902 ± 0.010
Main effects:	
Temperature (input A)	(−6.25 ± 20.2) × 10^−3^
Amount of DMSO (input B)	(−6.25 ± 20.2) × 10^−3^
Sonication time (input C)	(−6.25 ± 20.2) × 10^−3^
Effect of interaction:	
(input A) × (input B)	(−8.75 ± 20.2) × 10^−3^
(input A) × (input C)	(−8.75 ± 20.2) × 10^−3^
(input B) × (input C)	(−8.75 ± 20.2) × 10^−3^
(input A) × (input B) × (input C)	(6.25 ± 20.2) × 10^−3^

## Data Availability

The raw data supporting the conclusions of this article will be made available by the authors on request.
